# Serum protein gamma-glutamyl hydrolase, Ig gamma-3 chain C region, and haptoglobin are associated with the syndromes of pulmonary tuberculosis in traditional Chinese medicine

**DOI:** 10.1186/s12906-015-0686-4

**Published:** 2015-07-22

**Authors:** Ting-Ting Jiang, Chong Wang, Li-Liang Wei, Xiao-Mei Yu, Li-Ying Shi, Dan-Dan Xu, Zhong-Liang Chen, Ze-Peng Ping, Ji-Cheng Li

**Affiliations:** Institute of Cell Biology, Zhejiang University, Hangzhou, Zhejiang China; Department of Pneumology, The Sixth Hospital of Shaoxing, Shaoxing, Zhejiang China; Department of Clinical Laboratory, Zhejiang Hospital, Hangzhou, Zhejiang China

**Keywords:** Traditional Chinese Medicine Syndrome, Pathology, Erythrocyte sedimentation rate, Serum protein, Tuberculosis

## Abstract

**Background:**

Traditional Chinese Medicine (TCM) has been applied in treating tuberculosis (TB) based on the TCM syndromes with the effects of inhibiting Mycobacterium, strengthening the body immune system, and reducing the pulmonary toxicity. We used bioinformatic methods to study the clinical and pathological characteristics of pulmonary TB patients with TCM syndromes. Isobaric tags for relative and absolute quantification - coupled two dimensional liquid chromatography-tandem mass spectrometry (iTRAQ-2DLC-MS/MS) methods were applied to screen differentially expressed serum proteins.

**Methods:**

Pulmonary TB cases were divided into four distinctive TCM syndromes: pulmonary Yin deficiency (PYD) syndrome, hyperactivity of fire due to Yin deficiency (HFYD) syndrome, deficiency of Qi and Yin (DQY) syndrome, and deficiency of Yin and Yang (DYY) syndrome. The serum samples from 214 pulmonary TB patients were collected, and the clinical and pathological data was analyzed by using iTRAQ-2DLC-MS/MS. Finally, the differentially expressed proteins were screened and tested by ELISA. Only 5 patients with DYY syndrome were recruited in 3 years, which were not enough for further research.

**Results:**

The DQY cases had higher erythrocyte sedimentation rate (ESR) compared to the PYD and HFYD cases (*P* = 0.0178). 94.44 % (12 PYD, 18 HFYD, and 4 DQY before anti-TB treatment) of 36 treated TB cases were transformed to PYD accompanied with the reduction of ESR and absorption of pulmonary lesions. A total of 39 differentially expressed proteins (ratios of >1.3 or <0.75) were found among the three TCM syndromes. Proteomic studies revealed that gamma-glutamyl hydrolase (GGH), Ig gamma-3 chain C region (IGHG3), and haptoglobin (HPT) were specifically over-expressed in PYD (*P* < 0.01), HFYD (*P* < 0.001), and DQY cases (*P* < 0.01), respectively. Furthermore, GGH was significantly higher in PYD cases compared to the HFYD and DQY cases (*P* < 0.01, *P* < 0.001, respectively), whereas IGHG3 was significantly higher in HFYD cases than PYD and DQY cases (*P* < 0.001, *P* < 0.01, respectively).

**Conclusions:**

The results suggest that TCM syndromes are significantly correlated with the pulmonary lesions and ESR. GGH was associated with folate metabolism in PYD cases, IGHG3 was linked to the control of Mycobacterium infection in HFYD patients, and HPT was involved in hypoxia in DQY patients. The present study provides new biological basis to understand the pathological changes and proteomic differences of TB syndromes.

**Electronic supplementary material:**

The online version of this article (doi:10.1186/s12906-015-0686-4) contains supplementary material, which is available to authorized users.

## Background

Tuberculosis (TB) ranks as the second leading cause of death from an infectious disease, just after the AIDS. There were 8.6 million new TB cases and 1.3 million deaths including 0.3 million deaths from HIV-associated TB in 2012, globally [1]. The routine Western medicine for TB treatment has to last at least for 6 months, and may lead to drug-resistant TB [2] and severe hepatic side effect [3–5]. Traditional Chinese Medicine (TCM) has been applied in treating TB [6] based on the effects of inhibiting Mycobacterium, strengthening the body immune system, and reducing the pulmonary toxicity and drug-resistant. Chinese herbs such as *Gentiana rhodantha Franch* [7], *Astragalus membranaceus* [8], and *Radix Paeoniae Rubra (Chishao)* [9] have been described to be medicinally used for the treatment of TB. Other Chinese herbs such as *Radix Ranunculi Ternati*, *Radix Sophorae Flavescentis*, *Prunella Vulgaris L.* and *Stellera Chamaejasme L.* have been demonstrated to be effective in treating multi-drug resistant (MDR)-TB [6, 10]. These Chinese herbal medicines have either heat clearing and detoxifying or nourishing Yin and reducing fire effects. Biological researches revealed that *Astragalus membranaceus* extracts can strongly promote the phagocytosis of Mycobacterium [8]. *Radix Paeoniae Rubra* extracts can inhibit interleukin (IL)-10, and increase IL-8 in BCG-activated primary human blood macrophages [9]. IL-8 can attract T lymphocytes and neutrophils to the infection sites promoting the formation of granuloma at the early stage of Mycobacterium infection, and activating bactericidal response from neutrophils [11–13]. IL-10 is an anti-inflammatory cytokine produced by macrophages and T-cells during Mycobacterium infection [14]. Mycobacterium evades the host immunity with the help of IL-10 [15–17]. *Radix Paeoniae Rubra* extracts has been shown to inhibit the expression of IL-10, and can reduce the reactivation of TB and higher mycobacterial burden [18], thereby reducing the susceptibility to Mycobacterium infection [19]. However, the *Radix Sophorae Flavescentis*, *Radix Ranunculi Ternati*, *Stellera Chamaejasme L*. and *Prunella Vulgaris L*. extracts enhance cell-mediated immunity in multidrug resistant (MDR)-TB [10]. All these herbs can inhibit Mycobacterium and strengthen the body immune system.

The herbs in a TCM prescription for patients depend on the TCM syndrome differentiation [20]. Choosing suitable herbs and dosages according to the TCM syndromes differentiation would also improve the efficacy and reduce the toxic effects of anti-TB drugs [21, 22]. TCM syndrome, also called Zheng, is a temporary state at a period of time defined by symptoms, pulse feelings and tongue appearance [23]. The same disease can be classified into different syndromes. Differing from the Western medicine, the diagnosis of TCM syndrome is based on differentiation/classification [20]. TCM syndrome is assessed by inspection, auscultation, olfaction, interrogation, and palpation [20, 24]. Pulmonary TB patients were mainly divided into four TCM syndromes: pulmonary Yin deficiency (PYD) syndrome, hyperactivity of fire due to Yin deficiency (HFYD) syndrome, deficiency of Qi and Yin (DQY) syndrome, and deficiency of Yin and Yang (DYY) syndrome [25]. Yin-Yang (two interdependent, opposite, complementary, and exchangeable aspects of nature) have been employed by ancient Chinese scholars to analyze pulmonary TB [24]. Yin often refers to the material aspects of the organism, and Yang refers to function. In addition, Qi is another vital substance in TCM which has the function of promoting blood circulation in the body [24].

Disease occurs when the balance or harmony of Yin – Yang is disturbed [24]. PYD is the first stage of pulmonary TB, and the patients who have PYD syndrome can be characterized by Yin deficiency signs such as thirst, tussiculation, mild night sweat, and feverishness in palms and soles [25, 26]. HFYD is the second stage of pulmonary TB, and the patients who have HFYD syndrome can be characterized by Yin deficiency and fire hyperactivity signs such as steaming sensation in the bone, dysphoria with feverish sensation in chest, palms and soles [25, 26]. DQY is the third stage of pulmonary TB, and the patients are characterized by Yin and Qi deficiency signs such as weak and pale look. DYY is the last stage of the disease, and the patients who have DYY syndrome are almost in dead condition [25, 26].

However, criticism and skepticism was raised because the principles of TCM are rooted from ancient Chinese philosophy (Confucianism and Taoism), and syndrome differentiation depends heavily on subjective observation, clinical experience, and knowledge of practitioners with the lack of scientific researches and objective tests [24]. The current study employed bioinformatic methods (one-way ANOVA and *χ*2 test) to investigate clinical and pathological changes of the TCM syndromes of pulmonary TB, and to analyze the syndrome transformation after treatment. Isobaric tags for relative and absolute quantification (iTRAQ) - coupled with two dimensional liquid chromatography-tandem mass spectrometry (2DLC-MS/MS) was used to screen the specifically expressed serum proteins of pulmonary TB patients with distinctive TCM syndromes.

Our study is the first to analyze the pathological characteristics and proteomic differences of pulmonary TB in different TCM syndromes. We also provide solid pathological background and biological evidence for the TCM diagnosis.

## Methods

The present study was approved by the Medical Ethics Committee of Zhejiang University (China) and written informed consent was obtained from all the blood donors.

### Patients and control subjects

**Fig. 1:**
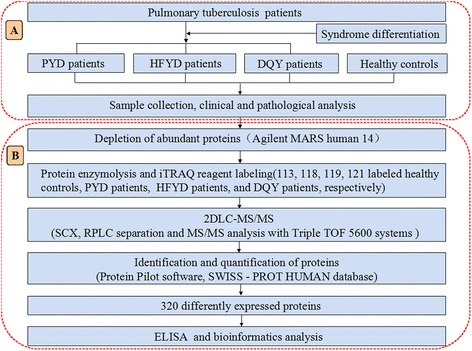
Schematic diagram of the experiment. **a** Diagnosed pulmonary TB patients were divided into four distinctive TCM syndromes (the number of DYY cases were insufficient for the experiment). The serum was collected, and the clinical and pathological characteristics were analyzed. **b** After removing abundant proteins, samples were denatured, alkylated, digested, and labeled with the iTRAQ tags as follows: healthy controls-113 isobaric tag, PYD syndrome-118 isobaric tag, HFYD syndrome-119 isobaric tag, DQY syndrome-121 isobaric tag. Samples were then separated and analyzed by 2DLC-MS/MS system, and differentially expressed proteins were further analyzed by ELISA and one-way ANOVA

The experimental design is illustrated in Fig. 1. A total of 214 pre-treated pulmonary TB cases (aged 18 to 75 years) and 36 TB-treated cases (aged 20 to 67 years) from the Sixth Hospital of Shaoxing (Shaoxing, Zhejiang, China) were included in the study. However, there were not enough DYY cases to be included in the investigation. In addition, 62 randomly chosen healthy blood donors, aged from 22 to 65 years, from the Zhejiang Hospital (Zhejiang, China) with no history of TB and other diseases were also included (Table 1).

**Table 1:** Characteristics of TB cases, treated-TB cases and healthy controls

	TB cases	Treated-TB cases	Healthy controls	*P* value
	(*N* = 214)	(*N* = 36)	(*N* = 62)	
Age, Age range (Mean ± SD)	18-75 (45.11 ± 15.08)	20-67 (44.33 ± 13.84)	22 − 65 (43.19 ± 8.84)	0.6337^a^
Gender (female: male)	72/142	10/26	30/32	0.8742^b^
Abnormal chest radiograph, no. (%)	214 (100)	2 (5.56)	ND	/
Positive sputum smears, no. (%)	152 (71.03)	0	ND	/
BCG vaccination, no. (%)	196 (91.59)	28 (82.35)	57 (91.94)	0.984^b^
Smoking, no. (%)	97 (45.33)	16 (44.44)	ND	/
HIV-negative, no. (%)	214 (100)	36 (100)	62 (100)	/

^a^ *P*-value between TB cases, treated-TB cases, and healthy controls, for one-way ANOVA

^b^ *P*-value between TB cases, treated-TB cases, and healthy controls for *χ* ^2^ test

*N*: number of subjects; ND: not determined

All pre-treated TB patients meet one of the following diagnostic criteria: 1. Positive sputum culture or smear; 2. Typical active TB findings on Chest X-ray and CT scan; 3. Pulmonary pathological lesions diagnosed as TB. Meanwhile, subjects with extra-pulmonary TB, diabetes, hepatitis B, AIDS and other diseases as well as immune inhibitor users were eliminated from the study. According to the “Standard of disease diagnosis and curative effect of Traditional Chinese Medicine”, all the cases were divided into PYD, HFYD, DQY, and DYY syndromes.

### Clinical and pathological analysis

Clinical data such as sputum culture and smear, tuberculin test, smoking history, BCG inoculation rate, ESR, blood coagulation, blood glucose level and pulmonary CT findings of PYD, HFYD, and DQY patients are shown in Table 2. The differences between PYD, HFYD, and DQY cases were analyzed by one-way ANOVA following Tukey post-hoc test and *χ*2 test using GraphPad Prism software. The transformation of TCM syndromes was also investigated after treatment.

**Table 2:** Characteristics of TB cases with PYD, HFYD and DQY Syndromes

	PYD (N = 71)	HFYD (N = 79)	DQY (N = 64)	*P* value
Age, Age range (Mean ± SD)	18-75 (43.36 ± 14.62)	18-75 (45.18 ± 15.08)	18-75 (46.78 ± 14.44)	0.4051^a^
Gender (female: male)	24/47	27/52	21/43	0.9924^b^
Positive sputum smears, no. (%)	50 (70.42)	53 (67.09)	49 (76.56)	0.4619^b^
Tuberculin skin test (>10 mm), no. (%)	40 (56.34)	47 (59.50)	41 (64.06)	0.6601^b^
Smoking, no. (%)	30 (42.25)	37 (46.84)	30 (46.88)	0.8188^b^
BCG vaccination, no. (%)	64 (90.14)	76 (96.20)	56 (87.5)	0.1537^b^
FIB(g/L) (Mean ± SD)	4.18 ± 1.11	3.82 ± 1.28	3.70 ± 1.15	0.0653^a^
PT(s) (Mean ± SD)	13.47 ± 1.45	13.15 ± 1.32	13.32 ± 1.27	0.3787^a^
APTT(s) (Mean ± SD)	31.20 ± 4.39	30.44 ± 4.60	30.27 ± 4.04	0.4464^a^
TT(s) (Mean ± SD)	14.42 ± 1.32	14.47 ± 1.13	14.78 ± 1.36	0.2483^a^
Blood glucose (mmol/L) (Mean ± SD)	4.90 ± 0.64	5.04 ± 0.55	5.11 ± 1.74	0.5184^a^
ESR (mm/h) (Mean ± SD)	11.15 ± 4.85	12.06 ± 5.91	13.72 ± 6.72	0.0388*^a^
	11.63 ± 5.44		13.72 ± 6.72	0.0178*^a^
CT scan				
Degenerative lesions: cavity, caseous necrosis	18 (25.35)	35 (44.30)	20 (31.25)	0.0427*^b^
Proliferative lesions	29 (40.85)	21 (26.58)	18 (28.13)	0.1307^b^
Exudation and other inflammatory lesions	21 (29.58)	20 (25.32)	17 (26.56)	0.8365^b^
Millet lesions	1 (1.41)	0 (0)	7 (10.94)	0.0013**^b^
Pleural lesion	2 (2.82)	3 (3.80)	2 (3.13)	0.9419^b^

^a^ *P*-value between TB cases with PYD, HFYD and DQY Syndromes for one-way ANOVA following Tukey post-hoc test

^b^ *P*-value between TB cases with PYD, HFYD and DQY Syndromes for *χ* ^2^ test

*N*: number of subjects; ND: not determined. *P < 0.05. **P < 0.01

### Serum collection and abundant protein depletion

Serum samples were collected in the vacuum tube without anticoagulation and centrifuged at 3000 rpm for 10 min at 4 °C. 10 serum samples from PYD, HFYD, DQY cases and healthy controls were mixed with the kit column (Agilent multiple affinity removal column LC column-14, the human MARS, Agilent, USA) to remove 14 highly abundant proteins, including albumin, IgG, antitrypsin, IgA, transferring, haptoglobin, fibrinogen, alpha 2-macroglobulin, alpha1-acid glycoprotein, IgM, apolipoprotein AI, apolipoprotein AII, complement C3, and transthyretin.

### Protein enzymolysis and iTRAQ labeling

The specimens were isotope-labeled according to the iTRAQ kit instructions (Applied Biosystem, Foster city, CA, USA). 100 μg of the samples from each group was mixed with pre-cooled acetone, and the pellet was dissolved with dissolution buffer and 1 % SDS. The reduced samples were alkylated and digested by trypsin (Sigma, St. Louis, MO, USA) overnight at 37 °C. Then the digested samples were iTRAQ labeled. The 113, 118, 119 and 121 isobaric tags were used to label the healthy controls, PYD, HFYD and DQY samples, respectively for 1 h at room temperature. The samples were obtained by vacuum centrifugal concentration (Christ RVC 2 to 25, Christ, Osterode, Germany) [27].

### 2D LC-MS/MS

The labeled samples were processed by Sep-Pak Vac C18 (Waters, Massachusetts, MA, USA) to remove the labeling reagent and salt. The labeled peptides were separated with strong cation exchange liquid chromatography column (2.1 mm × 100 mm, 5 μm, 200 A, Polysulfoethyl column, SCX) (Nest Group, Southborough, MA, USA). Buffer A (10 mM KH_2_PO_4_, 25 % ACN, PH2.6) and a linear gradient 0 %-80 % of buffer B (10 mM KH_2_PO_4_, 350 mM KCL, 25 % CAN, pH 2.6) was used to separate the samples at the flow rate of 200 μL /min for an hour [28]. A total of ten gradient fractions were collected for vacuum centrifugal concentration based on the peak type and time.

The samples were dissolved and loaded into the ZORBAX 300SB-C18 column (5 μm, 300 Å, 0.1 × 150 mm, Microm, Auburn, CA, USA). Each fraction was analyzed by Triple TOF 5600 system (Applied Biosystems) twice and exported as run 1, and run 2. The mass spectra were acquired by using positive ion mode and information-dependent acquisition mode (IDA). In IDA mode, survey scans were acquired between 400 to 1500 m/z, with up to 20 of the most intense multiply charged ions being sequentially chosen for MS/MS analysis. The mass range of product ion spectra was from 100 to 2000 m/z. The reporting iTRAQ ion intensities (113, 118, 119, and 121 m/z) were sufficiently enhanced for quantification.

### Proteins identification, quantification and bioinformatics analysis

Proteins identified and relatively quantified were performed by Protein Pilot software version 4.2 beta (Applied Biosystems) and International Protein Index database (version 3.87, HUMAN). We set the unused ProtScore >1.3 as cutoff to minimize false positives. More than 2 peptides with the 95 % confidence were chosen for quantification. Peptide quantification was also conducted by Pro Group algorithm to calculate *P* value, error factor, the reporter peak area, and to remove redundant hits. When the *P* value <0.05 and the error factor <2, the data was reliable [29]. Functional annotation and classification of proteins was analyzed by gene ontology (GO) database. Signaling pathways were conducted by using KEGG database. The protein-protein interaction was carried out by STRING software (http://string-db.org/). The fold changes ratios of >1.3 (up-regulated proteins) or <0.75 (down-regulated proteins) were chosen for further research.

### ELISA analysis

Differential proteins were measured in 154 TB cases (44 PYD cases, 55 HFYD cases, 55 DQY cases) and 62 healthy controls (randomly chosen) by ELISA. Human Haptoglobin ELISA kit (Abcam, London, England; the dilution was 1:2000), human IGHG3 ELISA kit (CUSABIO Biotech, Wuhan, Hubei, China; the dilution factor was 1:5000), and human GGH ELISA kit (CUSABIO Biotech, Wuhan, Hubei, China; the sample dilution was 1) were used to perform experiment in duplicates in accordance with the manufacturer’s instructions. The results were further analyzed by one-way ANOVA following Tukey post-hoc test. The study samples provided at least 83.57 % power to identify significant differences between TCM syndromes at a statistical support level of α = 0.05 with an effect size of 0.6 applying a two tails model calculated by Gpower3.0.5.

## Results

### Clinical and pathological analysis of Pulmonary TB cases

**Fig. 2:**
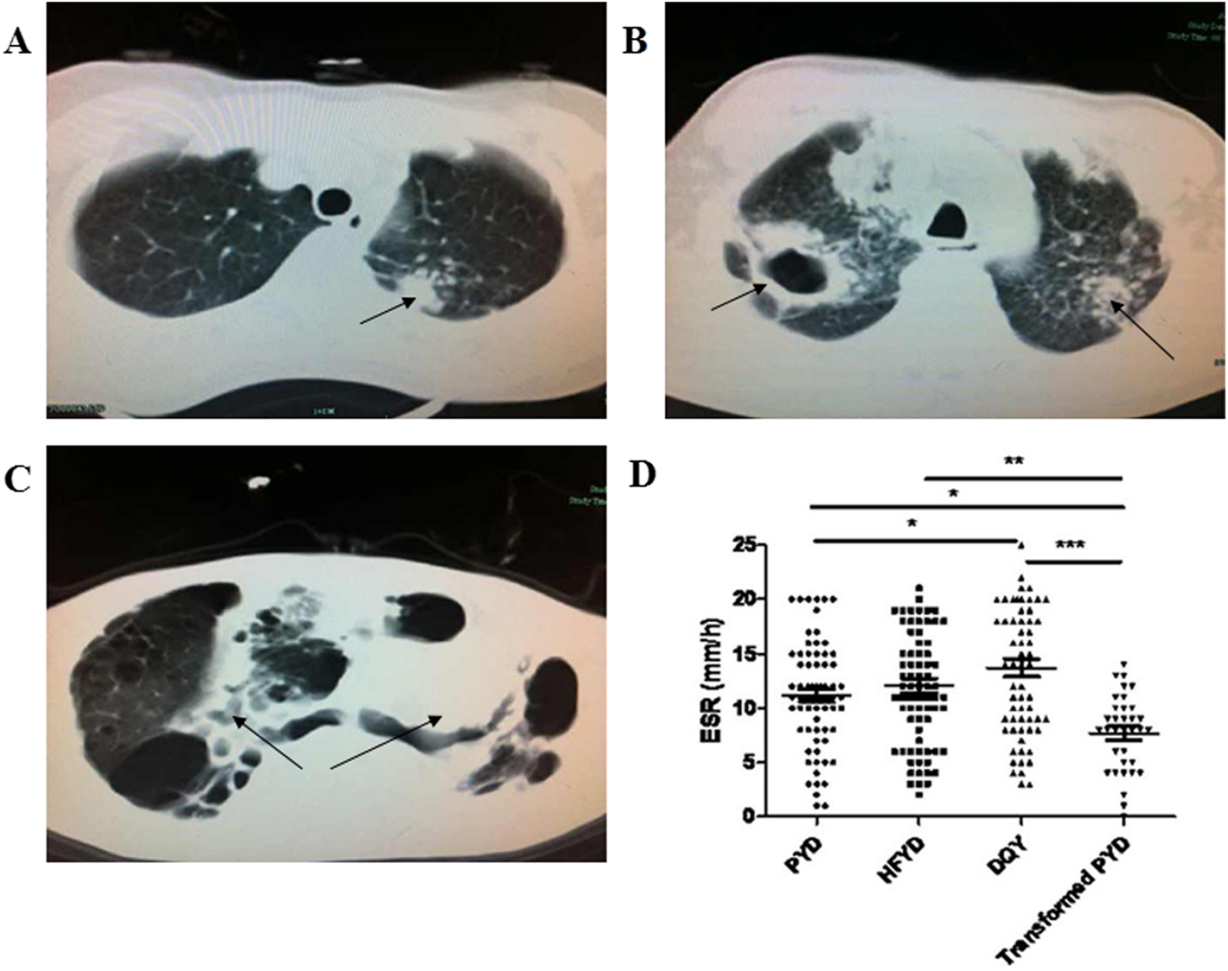
Radiographic CT findings and ESR analysis of pulmonary TB patients. **a** CT scan showing tubercular nodules (proliferative lesions); **b** CT scan showing pulmonary cavity and tubercular nodules (degenerative lesions and proliferative lesions); **c** CT scan showing multiple lesions including proliferative tuberculous nodules and fibroplastic pathological changes. **d** ESR characteristics of PYD, HFYD, DQY, and the transformed PYD patients. PYD: pulmonary Yin deficiency syndrome; HFYD: hyperactivity of fire due to Yin deficiency syndrome; DQY: deficiency of Qi and Yin syndrome; transformed PYD: TB patients who transformed to PYD syndrome after treatment. *P < 0.05, **P < 0.01, ***P < 0.001

The demographic characteristics of the TB patients, treated-TB patients and healthy controls are shown in Table 1. There were no significant differences between the TB patients, treated-TB patients, and healthy controls. The clinical symptoms and signs of TB cases with PYD, HFYD and DQY syndromes are described in Additional file 1. Statistical analysis was conducted by using GraphPad Prism software for the 71 PYD, 79 HFYD, and 64 DQY cases. CT scan findings could be divided into hyperplastic pulmonary lesions (tuberculous nodules, patch, stripping shadows), degenerative pulmonary lesions (empty and caseous necrotic changes), inflammatory lesions with leakages (flake, flocculent shadow and chronic inflammatory changes), pleural pulmonary lesions (pleural thickening and pleural effusion), and miliary TB. Chi-square analysis revealed that PYD cases had tuberculous nodules, patch and stripping shadows. HFYD cases were identified as having more degenerative pulmonary lesions, compared with the PYD and DQY cases. DQY cases had multiple pulmonary lesion areas with mixed pulmonary lesions and showed highest incidence of miliary TB, compared with the PYD and HFYD cases (Table 2, Fig. 2a−c). One-way ANOVA demonstrated that the ESR values were 11.15 ± 4.85 in PYD; 12.06 ± 5.91 in HFYD, and 13.71 ± 6.71 in DQY (*P* = 0.0388). Surprisingly, the ESR value was significantly higher in DQY, compared to the PYD and HFYD (*P* = 0.0178). The ESR value in HFYD was between PYD and DQY (Table 2, Fig. 2d).

### TCM transformation of TB cases

One-way ANOVA analysis of the 36 treated pulmonary TB cases revealed that after two to six months of treatment, 94.44 % (12 PYD, 18 HFYD, and 4 DQY before anti-TB treatment) of 36 treated TB cases were transformed to PYD accompanied with the reduction of ESR and absorption of pulmonary lesions. The ESR value was 7.70 ± 3.45, significantly lower than the ESR value of pre-treated cases (*P* < 0.0001), and the pulmonary cavity lesions were apparently absorbed and closed. However, 5.56 % of the cases (2 cases) were transformed into HFYD syndrome. The lesions were absorbed, but the pulmonary cavity lesions were not closed.

### Proteins identification and relative quantification

**Fig. 3:**
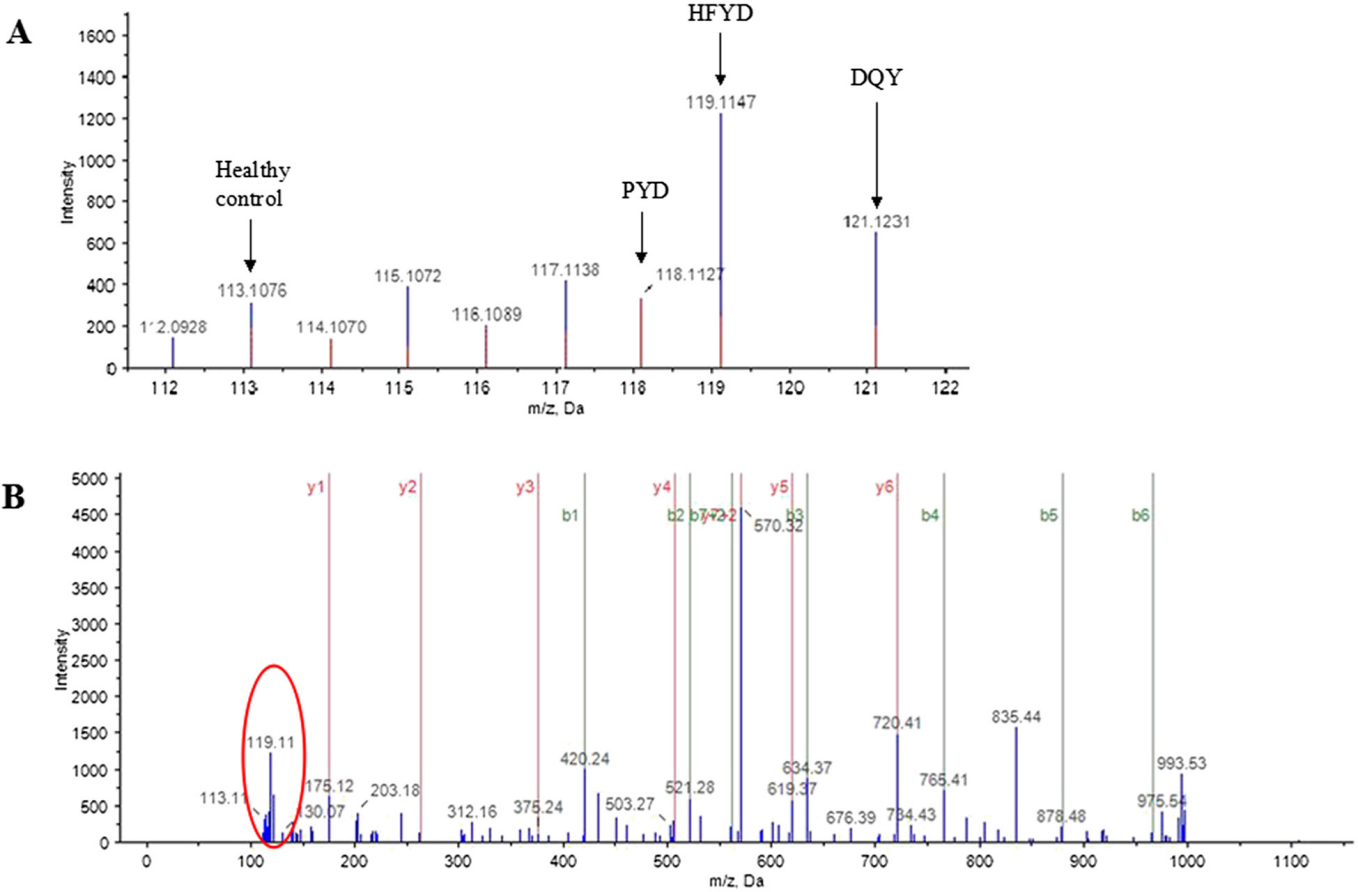
The representative MS/MS spectrum showed one of the peptides of IGHG3 protein. **a** The ion assignments were: 113-healthy control, 118-PYD syndrome, 119-HFYD syndrome, 121-DQY syndrome (114, 115, 116, 117 were for other diseases which were not included in this research). The intensity of ions from precursor peptides indicated protein expression levels. **b** The intensity of ions in MS/MS spectra showed identified peptide sequences with DTLMISR of IGHG3 protein

All iTRAQ labeled proteins were identified and relatively quantified. A total of 320 proteins were quantified two times in run 1 and run 2, with the unused ProtScore >1.3, error factor <2, and *P* < 0.05. The representative MS/MS spectrum of one peptide (DTLMISR) of IGHG3 protein is shown in Fig. 3. Among the 320 proteins, 116 proteins were identified in PYD with 64 up-regulated and 52 down-regulated proteins, and 108 proteins were identified in HFYD with 63 up-regulated and 54 down-regulated proteins. There were 96 proteins acquired in DQY with 51 up-regulated and 45 down-regulated proteins.

### Bioinformatic analysis

**Fig. 4:**
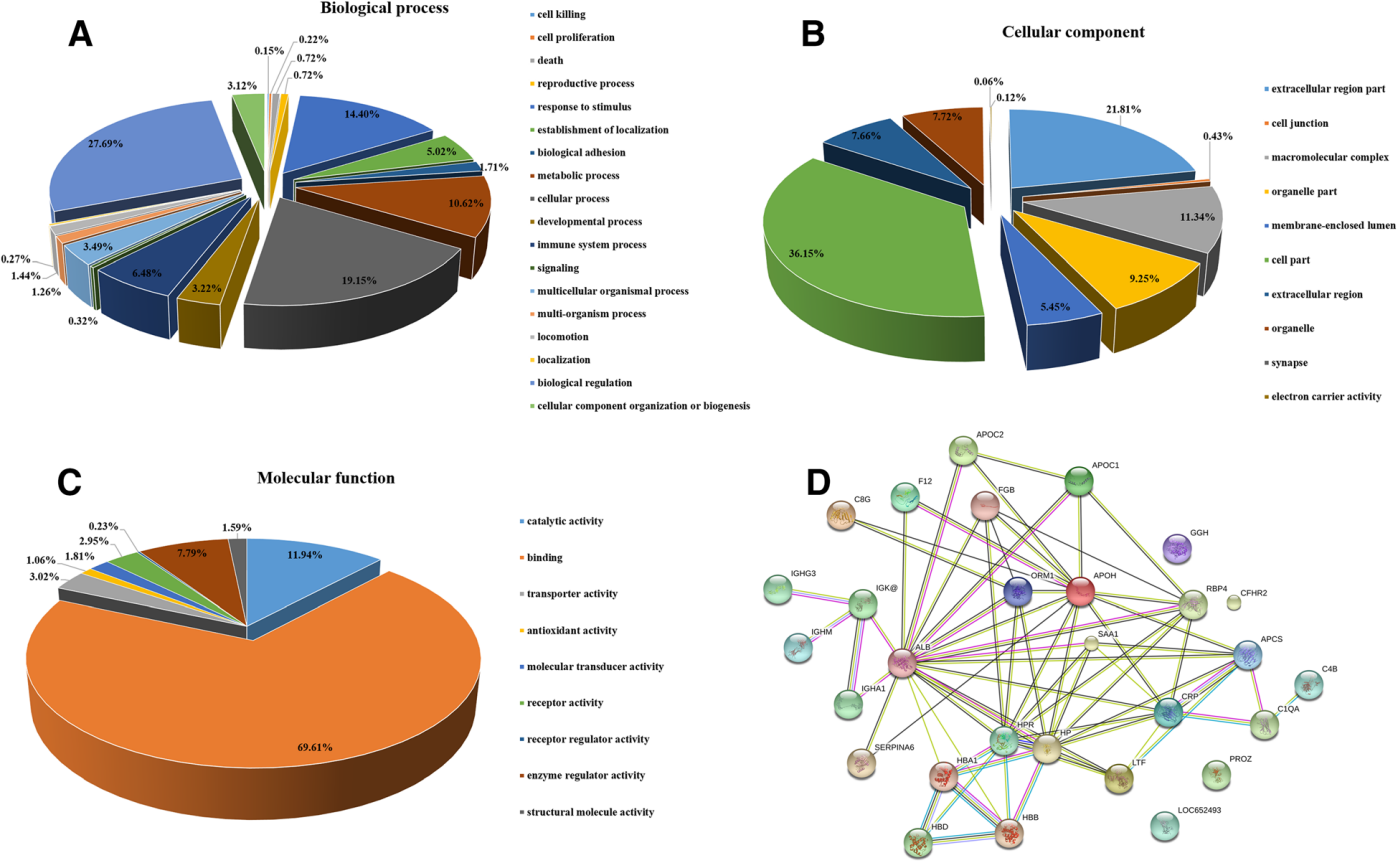
Data mining of differentially expressed proteins. **a** Biological process. **b** Cellular component. **c** Molecular function. **d** Functional network of differentially expressed proteins

For the statistical analysis among the three TCM syndromes, 39 differentially expressed proteins were found in the current study (Additional file 2). All these proteins were either over expressed (>1.3) or under expressed (<0.75) when compared with the healthy controls. GO analysis showed broad functional distribution for the 320 proteins, with the most frequently used categories of biological process, cellular component, and molecular function. It is indicated that most of the differential proteins were involved in the biological process of biological regulation (27.69 %), cellular process (19.15 %), response to stimulus (14.40 %), metabolic process (10.62 %), and immune system process (6.48 %). Notably, most over-expressed proteins of HFYD cases were involved in the immune system process (e.g., LAC2, IGHG3, IGHM, IGHA1, and IGLL5). In addition, most of the differentially expressed proteins were located in cell part (36.15 %) and extracellular region part (21.81 %). The analysis of molecular function suggested that most of the differentially expressed proteins have a role in binding (69.61 %) and catalytic activity (11.94 %) (Fig. 4). KEGG analysis indicated that the differential proteins participated in the complement clotting chain reaction (Additional file 3). Furthermore, by using functional network diagram analysis (STRING), it was found that 39 chosen proteins were involved in functional and physical connections (Fig. 4d).

### Verification of differentially expressed proteins by ELISA

**Fig. 5:**
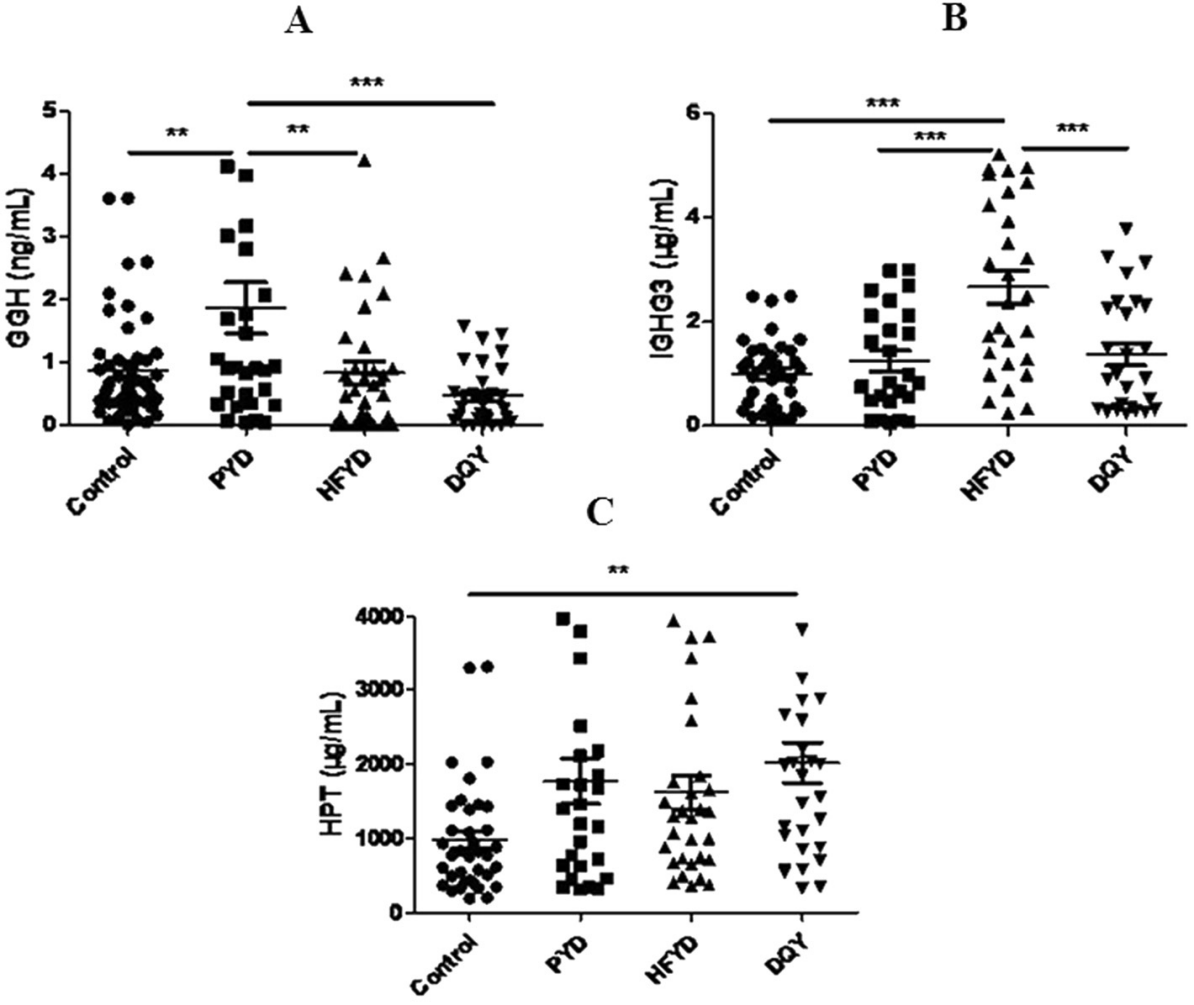
Analysis of differentially expressed proteins by ELISA and one-way ANOVA. **a** GGH; **b** IGHG3; **c** HPT. PYD: pulmonary Yin deficiency syndrome; HFYD: hyperactivity of fire due to Yin deficiency syndrome; DQY: deficiency of Qi and Yin syndrome; GGH: gamma-glutamyl hydrolase; IGHG3: Ig gamma-3 chain C region; HPT: haptoglobin. *P < 0.05, **P < 0.01, ***P < 0.001

By combining the differentially expressed protein ratios and bioinformatic analysis of GO, KEGG and STRING, significantly different proteins (ratios of >1.3 or <0.75) were chosen for ELISA analysis. All the proteins such as GGH, IGHG3 and HPT were closely related to the body’s metabolism, immune function and pulmonary TB. One-way ANOVA of the ELISA results showed a significant GGH increase in PYD cases compared with the healthy controls (*P* < 0.01, Fig. 5a). In HFYD cases, IGHG3 was significantly higher than the healthy controls (*P* < 0.001, Fig. 5b). In DQY cases, HPT was significantly higher than the healthy controls (*P* < 0.01, Fig. 5c). Comparisons between the three TCM syndromes revealed significant differences between GGH and IGHG3. GGH was significantly over-expressed in PYD cases compared with HFYD and DQY cases (*P* < 0.01, *P* < 0.001, respectively) (Fig. 5a). IGHG3 was specifically over-expressed in HFYD patients compared with PYD and DQY patients (*P* < 0.001, *P* < 0.01, respectively) (Fig. 5b).

## Discussion

We have studied the serum proteomics of TB cases with PYD, HFYD and DQY syndromes by SELDI–TOF MS combined with weak cation exchange (WCX) magnetic beads. However, a large amount of proteins cannot be screened and only one identified protein was acquired finally due to the limitation of the technology. The clinical and pathological analysis of PYD, HFYD and DQY cases was not sufficient [30]. We have also explored the specifically expressed serum proteins in TB patients by iTRAQ-2DLC-MS/MS, and found serum protein S100A9, SOD3, and MMP9 as new potential diagnostic biomarkers for pulmonary TB [31]. We applied the iTRAQ-2DLC-MS/MS technique to screen specifically expressed serum proteins in TB patients with distinctive TCM syndromes, and performed clinical and pathological analysis of specifically expressed proteins by bioinformatic methods.

Pulmonary TB occurs because of the disturbed balance between Yin and Yang [24]. Patients infected with Mycobacterium had Yin deficiency syndrome. With the progress of the disease, patients had severe imbalance in Yin-Yang, gradually leading to PYD and HFYD syndromes, and later on to the DQY syndrome. For those reaching the final stage of DYY syndrome, the patients were almost in dead condition.

### Clinical and pathological analysis

The clinical and pathological analysis showed that PYD cases basically had hyperplastic lesions, suggesting mild pathological changes. There were significantly more degenerative pulmonary lesions in the HFYD cases, compared with PYD and DQY cases. Degenerative lesions were due to the strong delayed-type hypersensitivity (DTH), indicating allergic reaction [32]. However, DQY cases have multiple lesions, often accompanied by a variety of pathological changes, including miliary lesions that was much higher than the PYD and HFYD cases (*P* = 0.0013), suggesting severe TB.

The ESR value in the TB cases showed an interesting phenomenon: it was significantly higher in DQY patients, compared to the PYD and HFYD patients (*P* = 0.0178, Table 2, Fig. 2d). ESR could reflect TB severity. The higher the ESR value, the severe the pathological stages of TB. In the late pulmonary TB stages, ESR reached the highest [33, 34]. So, the DQY syndrome belongs to the most severe stage with highest ESR value among the syndromes. Thus, it is believed that TCM syndromes of TB may be closely related to the ESR and pathological lesions.

The results revealed that 94.44 % of 36 treated TB cases (12 PYD, 18 HFYD, and 4 DQY before anti-TB treatment) were transformed into PYD with obvious TB lesion absorption, closing of the pulmonary cavity lesions, negative sputum smear/culture and improved physical signs, and significantly reduced ESR, compared with the pre-treatment cases. Only 5.56 % of the cases (2 cases) showed HFYD syndrome, and the pulmonary cavity remained unclosed. The ESR reduced, but with no statistical significance due to the few case numbers. All the data pointed to the fact that the treatment changed the TCM syndromes to PYD with improved signs and symptoms.

The current study employed healthy donors with balanced Yin-Yang as the controls. Serum proteomics was investigated in the three TCM syndromes by iTRAQ-2DLC-MS/MS. The results showed that TCM syndromes were mostly associated with GGH, IGHG3, and HPT proteins. GGH was specifically over-expressed in the PYD syndrome involved in the metabolism; IGHG3 was significantly over-expressed in the HFYD syndrome for the immune status; and HPT was specifically over-expressed in the DQY syndrome participating in the biological regulations. We suggested that all these proteins may be involved in the TB pathogenesis with close correlation to the TCM syndromes. ELISA analysis supported the serum proteomics findings.

### GGH was associated with PYD syndrome of Pulmonary TB

The results showed that GGH in the PYD cases was significantly higher than the healthy controls. GGH could yield pteroyl-alpha- glutamate (folate) and free glutamate through hydrolysis, and exert an important role in the bioavailability of dietary pteroylpolyglutamates and in the metabolism of pteroylpolyglutamates and antifolates [35, 36]. The change in serum GGH concentration could also change the intracellular or extracellular folate concentration [37]. Folate is necessary for the survival and toxicity of Mycobacterium. It is also an important cofactor for DNA synthesis and repair during Mycobacterium proliferation [38, 39]. Mycobacterium must synthesize folate themselves, because they could not use the exogenous folate directly. The host could provide materials for the folate synthesis. So, we speculate that GGH may affect the folic acid intake of Mycobacterium by affecting the body’s folate metabolism. In PYD cases, when GGH increased, the body could make better use of the folate from food. Excessive folate can produce more glutamate and para-aminobengoic acid as synthesizing material for the folate synthesis. It was also found that GGH in PYD cases was much higher than in the HFYD and DQY cases, indicating that folate was most effectively used in PYD cases. Thus, serum GGH may be associated with folic acid intake of Mycobacterium in PYD patients.

### IGHG3 was associated with HFYD syndrome of Pulmonary TB

In this study, IGHG3 in the HFYD cases was significantly higher than the healthy controls. IGHG3 could bind with IgG Fc receptor (FcγR) of neutrophilic granulocyte and macrophages, thereby promoting phagocytosis and controlling the infection [40]. IGHG3 could also combine with FcγRI on the surface of dendritic cells with high affinity, and enhance the antigen presentation function of dendritic cells to T lymphocyte [41–43]. Elevated serum IGHG3, on the one hand could combine with FcγR, mediate the macrophage phagocytosis, and control the infection. On the other hand, increased IGHG3 could be combined with FcγRI so as to increase the antigen presenting function of dendritic cells. It was also observed that IGHG3 in the HFYD cases were much higher than the PYD and DQY cases. The current results suggested that IGHG3 may be associated with the control of Mycobacterium infection in HFYD patients. In clinical practice, HFYD has obvious symptoms of internal fire due to Yin deficiency, presenting fever, while in PYD and DQY cases, fever signs were not so evident. The relationship between IGHG3 and the internal fire of HFYD syndrome need further research.

### HPT was associated with DQY syndrome of Pulmonary TB

In this experiment, HPT in the DQY cases was significantly higher than the healthy controls. HPT could capture and combine with free hemoglobin, while hemoglobin could carry oxygen from the lung surface to the aerobic tissues. In clinical practice DQY patients have obvious symptoms of hypoxia, such us cyanosis. Hypoxia is sequelae of TB and concomitant pulmonary diseases [44]. TB may even lead to respiratory distress syndrome with more severe hypoxia [45, 46]. When the tissue is in a state of hypoxia, it will increase the oxygen demand and change the hemoglobin by configuration so as to improve the oxygen usage [47]. But increased hemoglobin could promote the production of oxygen radicals, leading to oxidative damage and cell death [48–50]. HPT can combine with hemoglobin so as to stop the production of oxygen radicals in the blood and its toxic effects [51, 52]. While in PYD and HFYD cases, its value was between the healthy controls and DQY cases. The results were in correspondence with the clinical syndromes and TCM theories of TB cases. Increased HPT may indicate hypoxic condition and was closely associated with the DQY syndrome in TB patients.

In order to amplify the value of our study, a larger number of TB patients with DYY syndrome should be further tested. Further studies with treated patients are needed to confirm the importance of these results as potential biomarkers of PYD, HFYD and DQY syndromes.

## Conclusions

To sum up, TCM syndromes classification in pulmonary TB cases were closely related to the clinical and pathological changes and ESR. After treatment, most cases were transformed into PYD syndrome. Serum proteins such as GGH, IGHG3 and HPT were specifically over-expressed in PYD, HFYD, and DQY patients, respectively. GGH was associated with folate metabolism in PYD patients, and IGHG3 was linked to the control of Mycobacterium infection in HFYD patients. However, HPT was involved in hypoxia in DQY patients. The results provide clinical evidence and experimental basis for TCM syndromes of pulmonary TB. In addition, the data laid biological basis for the TCM syndromes classification, especially, providing an important clue for TCM differential diagnosis with improved and important scientific significance.

## Additional files

Additional file 1:
**Clinical symptoms and signs of TB cases with PYD, HFYD and DQY syndromes.** PYD: pulmonary Yin deficiency syndrome. HFYD: hyperactivity of fire due to Yin deficiency syndrome. DQY: deficiency of Qi and Yin syndrome. Additional file 2:
**Differentially expressed proteins and their expression levels quantified by iTRAQ-2DLC-MS/MS analysis.** iTRAQ ratio value: the fold changes ratio of proteins between different groups. Unused ProtScore: >1.3 was set as cutoff for protein identification. % Cov (95): the coverage of peptide with the 95 % confidence. Additional file 3:
**KEGG analysis chart of differentially expressed proteins.** Most of the proteins were involved in Complement activation and coagulation cascades pathway.
