# Supplementary figures and images for: Serum protein gamma-glutamyl hydrolase, Ig gamma-3 chain C region, and haptoglobin are associated with the syndromes of pulmonary tuberculosis in traditional Chinese medicine
Source: BMC Complement Altern Med. 2015 Jul 22;15:243. doi: 10.1186/s12906-015-0686-4 (PMC4509701; doi:10.1186/s12906-015-0686-4)

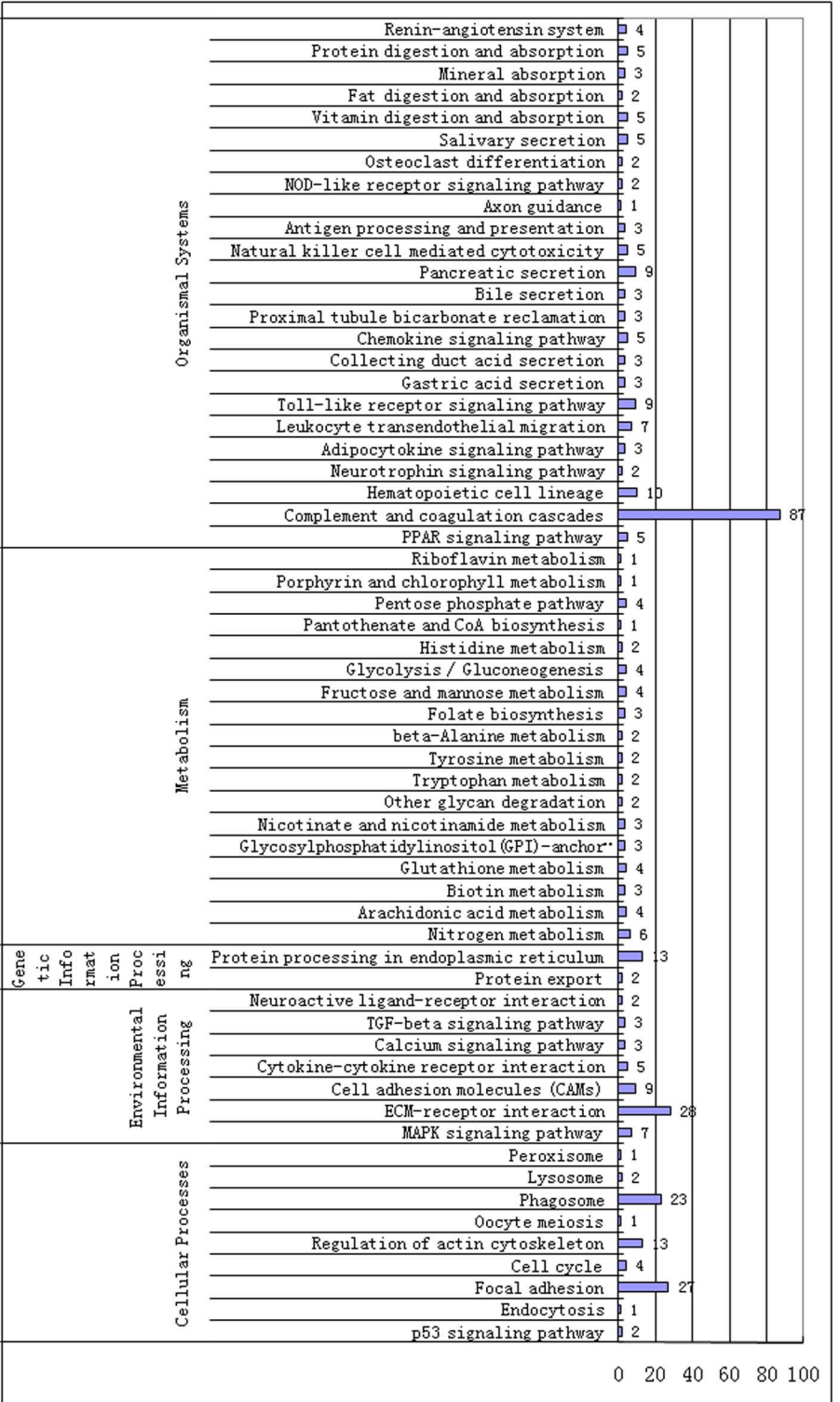

Supplement: Additional file 3: — KEGG analysis chart of differentially expressed proteins. Most of the proteins were involved in Complement activation and coagulation cascades pathway. [file 12906_2015_686_MOESM3_ESM.pdf]
